# Visualization of secretory cargo transport within the Golgi apparatus

**DOI:** 10.1083/jcb.201807194

**Published:** 2019-03-11

**Authors:** Kazuo Kurokawa, Hiroko Osakada, Tomoko Kojidani, Miho Waga, Yasuyuki Suda, Haruhiko Asakawa, Tokuko Haraguchi, Akihiko Nakano

**Affiliations:** 1 Live Cell Super-Resolution Imaging Research Team, RIKEN Center for Advanced Photonics, Saitama, Japan; 2 Advanced ICT Research Institute Kobe, National Institute of Information and Communications Technology, Kobe, Japan; 3 Department of Chemical and Biological Sciences, Faculty of Science, Japan Women’s University, Tokyo, Japan; 4 Laboratory of Molecular Cell Biology, Faculty of Medicine, University of Tsukuba, Tsukuba, Japan; 5 Graduate School of Frontier Biosciences, Osaka University, Suita, Japan

## Abstract

To describe trafficking of secretory cargo within the Golgi apparatus, the cisternal maturation model predicts that Golgi cisternae change their properties from cis to trans while cargo remains in the cisternae. Cisternal change has been demonstrated in living yeast *Saccharomyces cerevisiae*; however, the behavior of cargo has yet to be examined directly. In this study, we conducted simultaneous three-color and four-dimensional visualization of secretory transmembrane cargo together with early and late Golgi resident proteins. We show that cargo stays in a Golgi cisterna during maturation from cis-Golgi to trans-Golgi and further to the trans-Golgi network (TGN), which involves dynamic mixing and segregation of two zones of the earlier and later Golgi resident proteins. The location of cargo changes from the early to the late zone within the cisterna during the progression of maturation. In addition, cargo shows an interesting behavior during the maturation to the TGN. After most cargo has reached the TGN zone, a small amount of cargo frequently reappears in the earlier zone.

## Abstract (precis)

Kurokawa et al. visualize the transport of secretory cargo in the Golgi apparatus in living yeast cells. Cargo stays in the cisterna, whose property changes from cis to trans and further to the trans-Golgi network, but shows a dynamic behavior between the early and the late zones within the maturing cisterna.

## Introduction

In the secretory pathway, secretory cargo proteins newly synthesized in the ER are delivered to the Golgi apparatus, where they are processed and glycosylated before being sorted to their final destinations (Mellman and Warren, 2000; Emr et al., 2009). The Golgi apparatus is the central station of membrane traffic and is usually in the form of ordered stacks of several cisternae. Secretory cargo travels across the stack from the cis side to the trans side of cisternae and then to the trans-Golgi network (TGN; Griffiths and Simons, 1986). The mechanism of cargo trafficking within the Golgi apparatus has been a hot issue in the field of membrane trafficking (Emr et al., 2009; Glick and Nakano, 2009; Nakano and Luini, 2010; Glick and Luini, 2011). Three major models of anterograde cargo trafficking have been proposed: (a) traffic via cisternal maturation, (b) traffic by anterograde vesicular carriers, and (c) traffic via interconnected continuity of cisternae (Pfeffer, 2010; Glick and Luini, 2011). Which of these mechanisms best explains the intra-Golgi cargo trafficking still remains controversial. For example, recent two studies using inducible unnatural protein aggregation have led to opposite conclusions (Lavieu et al., 2013; Rizzo et al., 2013).

Among these models, the cisternal maturation model has been favored to explain the live imaging observation in yeast that Golgi cisternae change their properties over time (Losev et al., 2006; Matsuura-Tokita et al., 2006; Rivera-Molina and Novick, 2009) and the movement of large cargo through the stacks without leaving cisternae in mammals (Bonfanti et al., 1998; Lanoix et al., 2001; Martinez-Menárguez et al., 2001; Mironov et al., 2001). The original cisternal maturation model assumed that Golgi cisternae newly form, progressively mature, and finally dissipate. Secretory cargo is predicted to remain in the maturing Golgi cisterna. What was lacking in the proof of this mechanism is the demonstration of cargo delivery in living cells. Because the Golgi cisternae of the budding yeast *Saccharomyces cerevisiae* are not stacked but are scattered in the cytoplasm, their change from cis to trans nature was relatively easy to observe, but the behavior of cargo during this process remained elusive. Now we have succeeded in simultaneous three-color and 4D observation to visualize a transmembrane secretory cargo together with the early and late Golgi resident proteins by the high-speed and high-resolution microscopic technology we developed, super-resolution confocal live imaging microscopy (SCLIM; Kurokawa et al., 2013). We show, in *S. cerevisiae*, that transmembrane secretory cargo stays within the Golgi cisterna that contain both the early and late zones, but in a dynamic way. We often observe apparent backward relocation behavior of cargo from the TGN to an earlier zone.

## Results

### Three-color 4D observation visualizes sequential unidirectional maturation from cis-Golgi to trans-Golgi and then to the TGN

For the analysis of behaviors of Golgi cisternae in yeast, a variety of pairs of Golgi markers residing in early and late Golgi cisternae have been used as fusions with fluorescent proteins (Losev et al., 2006; Matsuura-Tokita et al., 2006; Rivera-Molina and Novick, 2009; Suda et al., 2013). To quantitate the rate of “maturation,” the transition times between the intensity peaks for each pair of Golgi markers on a single cisterna (peak-to-peak time; Daboussi et al., 2012) and the colocalization rates between the Golgi markers have been analyzed (Ishii et al., 2016).

**Figure 1.**
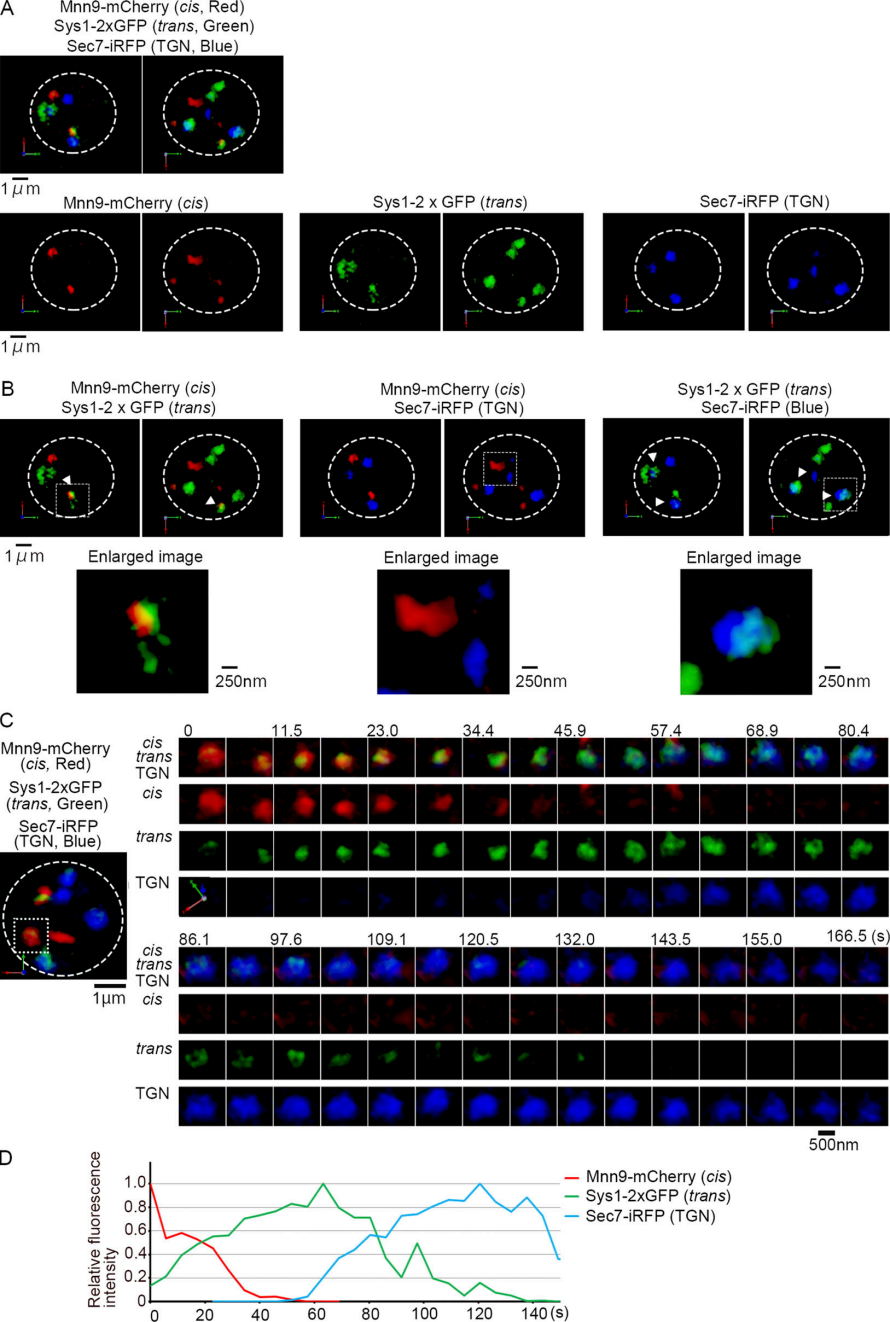
**Three-color 4D observation of Golgi cisternal maturation in living yeast cells.** WT cells expressing Mnn9-mCherry (cis-Golgi, red), Sys1-2xGFP (trans-Golgi, green), and Sec7-iRFP (TGN, blue) were observed by SCLIM. **(A)** Representative 3D images of merged (cis, trans, and TGN markers, upper) and each marker (lower) are shown. Scale bar, 1 µm. **(B)** 3D merged images of cis and trans, cis and TGN, and trans and TGN markers are shown (upper). Scale bar, 1 µm. Lower panels show enlarged images of selected cisternae (boxed areas in upper panels). Scale bar, 250 nm. **(C)** A representative 3D image of cisternae is shown on the left. Scale bar, 1 µm. Right panels show 4D (3D time-lapse) images of a selected cisterna (boxed area in the left panel). 3D images were reconstructed from 31 optical slices 100 nm apart around the center of the cell taken at 6.25-s intervals. Merged (cis, trans, and TGN markers) and each marker’s images are shown. Scale bar, 500 nm. **(D)** Relative fluorescence intensity changes of cis, trans, and TGN markers in the selected cisterna in C are shown. Statistical data are shown in Fig. 2 C.

**Figure 2.**
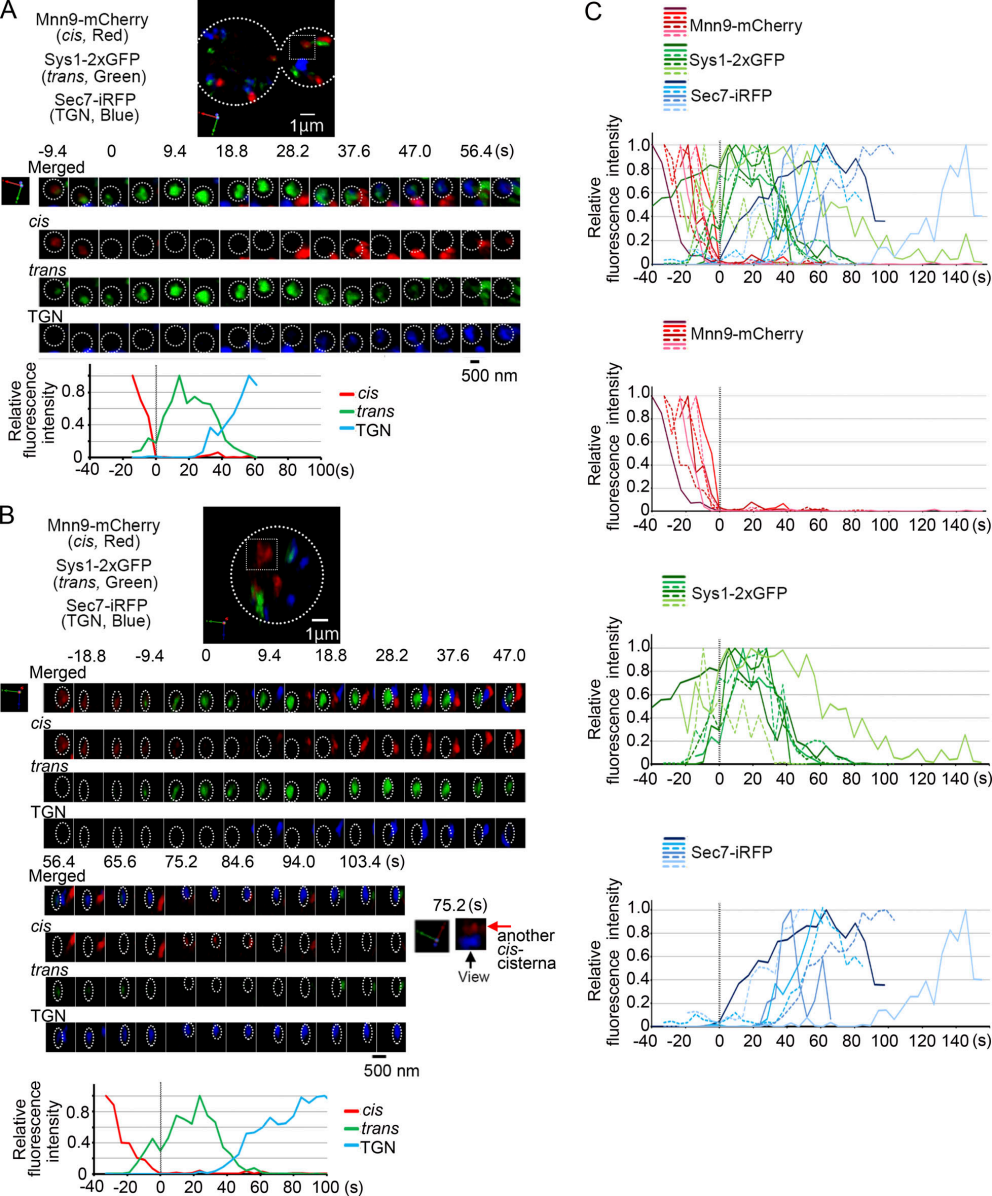
**Dynamics of three Golgi markers Mnn9-mCherry (cis-Golgi), Sys1-2xGFP (trans-Golgi), and Sec7-iRFP (TGN).** WT cells expressing Mnn9-mCherry (cis-Golgi, red), Sys1-2xGFP (trans-Golgi, green), and Sec7-iRFP (TGN, blue) were observed by SCLIM. **(A and B)** Representative 3D images of cisternae are shown on the top. Scale bar, 1 µm. Middle panels show 4D (3D time-lapse) images of selected cisternae (boxed area in the top panels). 3D images were reconstructed from 31 optical slices 200 nm apart around the center of the cell taken at 4.7-s intervals. The frames where Mnn9-mCherry disappeared were set as time 0. Merged (cis, trans, and TGN markers) and each marker’s images are shown. The cis-Golgi signals seen at ∼75.2 s in B were due to the fluorescence of another cis-Golgi cisterna behind the selected cisterna as shown by the 3D-rotated image (rightmost). Scale bar, 500 nm. Lower panels show relative fluorescence intensity changes of cis, trans, and TGN markers in the selected cisternae in middle panels. **(C)** Compiled relative fluorescence intensities of three markers (top), Mnn9-mCherry (second), Sys1-2xGFP (third), and Sec7-iRFP (bottom) over time from seven independent cisternae are shown. The frames where Mnn9-mCherry signals disappeared were set as time 0.

In our previous studies, we used Sys1, a receptor for Arl3, and Sec7, a guanine-nucleotide exchange factor for Arf GTPases, as markers for trans-Golgi cisternae and the TGN (Achstetter et al., 1988; Behnia et al., 2004; Ishii et al., 2016). However, we realized that the localization patterns of these two proteins were quite different. The percentage colocalization of Mnn9-mCherry with Sys1-GFP was much higher than that with Sec7-GFP (Ishii et al., 2016). We also found that Sec7 colocalized very well with clathrin and clathrin adaptor proteins (AP-1 and Gga) but rarely overlapped with COPI (coat protein complex I) coat proteins, whereas Sys1 showed good colocalization with COPI but not with clathrin (unpublished data). These findings prompted us to redefine Sec7 as a marker for the TGN, not for the trans cisternae, and Sys1 as a more appropriate marker for trans-Golgi. In the present study, we constructed a yeast strain constitutively expressing three Golgi markers: Mnn9-mCherry residing in cis-Golgi, Sys1-2xGFP for trans-Golgi, and Sec7-iRFP for the TGN (Jungmann and Munro, 1998; Behnia et al., 2004; Shu et al., 2006; Filonov et al., 2011). Simultaneous three-color observation of this strain by SCLIM exhibited dispersed localizations of Mnn9-mCherry–, Sys1-2xGFP–, and Sec7-iRFP–positive cisternae in the cytoplasm (Fig. 1 A), reflecting preferential distribution of Golgi markers to particular cisternae. We found some cisternae colabeled with Mnn9-mCherry and Sys1-2xGFP (Fig. 1 B, arrowheads in left panels) and others with Sys1-2xGFP and Sec7-iRFP (Fig. 1 B, arrowheads in right panels), whereas Mnn9-mCherry and Sec7-iRFP dual-positive cisternae were rarely observed (Fig. 1 B, central panels) as we reported previously (Ishii et al., 2016). The three-color 4D SCLIM observation of this strain (Fig. 1, C and D; and Fig. 2, A and B) demonstrated that a cis-Golgi cisterna labeled with Mnn9-mCherry (red) changed its color to Sys1-2xGFP positive (green), and then, after most of Mnn9-mCherry departed this cisterna, changed into the TGN labeled with Sec7-iRFP (blue; see also Video 1). We have previously shown the kinetics of cisternal maturation from cis to medial and then to trans in more detail (Ishii et al., 2016). The medial marker is always in between cis and trans markers and is thus not dealt with in this paper. Note that different Golgi markers show segregation within single mixing cisternae (Fig. 1, B [enlarged images] and C) as recognized before (Matsuura-Tokita et al., 2006; Ishii et al., 2016). To understand the kinetics of cisternal maturation of the Golgi, we analyzed the fluorescence intensities of these three markers in seven independent cisternae by setting the frame where Mnn9-mCherry signal disappeared as time 0 (Fig. 2 C). Sec7-iRFP signals emerged always after 0 s, indicating that cisternae change its nature to the TGN after Mnn9 has departed. All the Sys1-2xGFP signals had overlapping time periods, earlier with Mnn9-mCherry and later with Sec7-iRFP signals.

### Golgi markers localized to different zones are present within the continuous membrane structure

**Figure 3.**
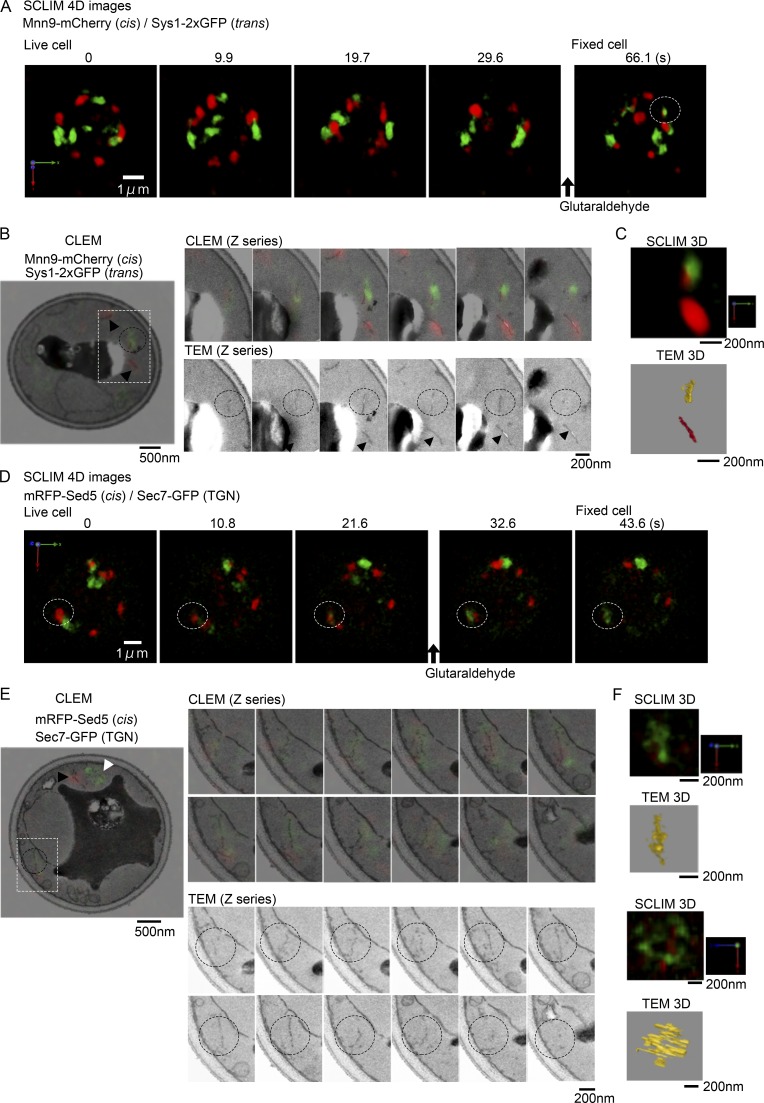
**Live CLEM analysis of maturing Golgi cisternae harboring different markers. (A)** 3D images of WT cells expressing Mnn9-mCherry (cis-Golgi, red) and Sys1-2xGFP (trans-Golgi, green) were reconstructed from 61 optical slices 100 nm apart taken at 9.85-s intervals. A white dotted circle indicates a cisterna maturing from cis to trans, in which cis- and trans-Golgi markers appeared to localize in a spatially segregated fashion. Glutaraldehyde was added at 30.0 s, and the image of the fixed cell was obtained at 66.1 s. Scale bar, 1 µm. **(B)** CLEM images of the cell indicated in A. Left panel shows a section image of fluorescence signals superimposed with the TEM image of the corresponding section. Black arrowheads indicate cis-Golgi cisternae. Black dotted circles show the same cisterna as indicated by the white dotted circle in A. Scale bar, 500 nm. Right panels show serial sections of CLEM (upper) and TEM (lower) images of selected cisternae (boxed area in the left panel). Scale bar, 200 nm. **(C)** Enlarged 3D reconstructed images of the maturing cisterna and a cis-Golgi cisterna by SCLIM (upper) and TEM (lower). The yellow structure in TEM 3D indicates the maturing Golgi cisterna, and the red indicates a neighboring cis-Golgi cisterna. Continuous membrane structure was observed in all of three independent maturing cisternae. Scale bar, 200 nm. Full 3D reconstruction of the SCLIM and TEM images is animated in Video 2. **(D)** 3D images of WT cells expressing mRFP-Sed5 (cis-Golgi, red) and Sec7-GFP (TGN, green) were reconstructed from 81 optical slices 80 nm apart of cell taken at 10.8-s intervals. White dotted circles indicate a maturing cisterna from cis to TGN, whose markers appeared to localize in a segregated fashion. Glutaraldehyde was added after 21.6 s, and the cell was judged to be fixed between 32.6 and 43.6 s. Scale bar, 1 µm. **(E)** CLEM images of the cell indicated in D. Left panel shows the section image of fluorescence signals superimposed with the TEM image of the corresponding section. Black and white arrowheads indicate cis-Golgi and TGN cisternae, respectively. Black dotted circles show the same cisterna indicated by the white dotted circle in D. Scale bar, 500 nm. Right panels show serial sections of CLEM (upper) and TEM (lower) images of the selected cisterna (boxed area in the left panel). Scale bar, 200 nm. **(F)** 3D reconstructed images of the maturing cisterna by SCLIM (upper) and TEM (upper second). Lower two images indicate the same 3D reconstructed images shown at another angle. Scale bar, 200 nm. Full 3D reconstruction of SCLIM and TEM images is animated in Video 3.

Segregation of different Golgi markers within a mixing Golgi cisterna suggests that different zones remain during cisternal maturation. To validate whether the zones labeled with different Golgi markers are present in the continuous membrane structure of the Golgi, we next conducted live-cell imaging followed by correlative light and electron microscopy (live CLEM; Asakawa et al., 2010, 2014). First, using dual-color 4D SCLIM, we observed the yeast cells that express Mnn9-mCherry and Sys1-2xGFP or mRFP-Sed5, a soluble *N*-ethylmaleimide-sensitive factor attachment protein receptor (SNARE) molecule residing mainly in the cis-Golgi, and Sec7-GFP (Fig. 3, A and D; Wooding and Pelham, 1998; Campbell et al., 2002). Cisternal maturation from cis-Golgi to the TGN was monitored by using the pair of markers Sed5 and Sec7 because Sed5 stays longer on the maturing cisterna than Mnn9 and covers later stages as well (Matsuura-Tokita et al., 2006; Ishii et al., 2016). During fluorescence observation, glutaraldehyde was added at the indicated times to fix the cells (Fig. 3, A and D). After fixation, bright-field and vacuolar intrinsic fluorescence images were taken as references for correlation with transmission electron microscopy (TEM) images (Fig. S1), and then the cells were postfixed with KMnO_4_ and embedded in resin. Thin sections of the resin block were cut, and the cells observed by 4D SCLIM were analyzed by TEM. The section images of SCLIM fluorescence were superimposed with the TEM images of the corresponding section. Individual membrane structures of Golgi cisternae were identifiable in TEM images by correlation with fluorescence images of Golgi markers (Fig. 3, B and E). A 3D tomographic reconstruction image of TEM corroborated that a maturing Golgi cisterna, in which Mnn9-mCherry (cis-Golgi marker) and Sys1-2xGFP (trans-Golgi marker) appeared to locate in a segregated way, had an apparently continuous membrane structure (Fig. 3, A [dotted circle at 66.1 s] and C; and Video 2, yellowish structure). Other cis-Golgi cisternae were also confirmed to be almost continuous membrane structures (Fig. 3, B [black arrowhead] and C; and Video 2, red structure). Fig. 3 D demonstrates that a cisterna in transition into the TGN (dotted circle at 43.6 s), containing a small zone labeled with cis-Golgi marker (mRFP-Sed5) while the rest comprising a large zone labeled with TGN marker (Sec7-GFP), is in a continuous fenestrated membrane structure (Fig. 3 F and Video 3, yellowish structure). Thus, the reconstructed 3D images of Golgi cisternae by SCLIM are very well correlated with the membrane structures demonstrated by tomographic reconstruction of TEM images (Fig. 3, C and F; and Videos 2 and 3). These results indicate that segregated zones marked with different fluorescent proteins in the Golgi are indeed in the continuous membrane structure of a Golgi cisterna.

### Secretory cargo is transported from cis- to trans-Golgi while being maintained within a maturing cisterna

**Figure 4.**
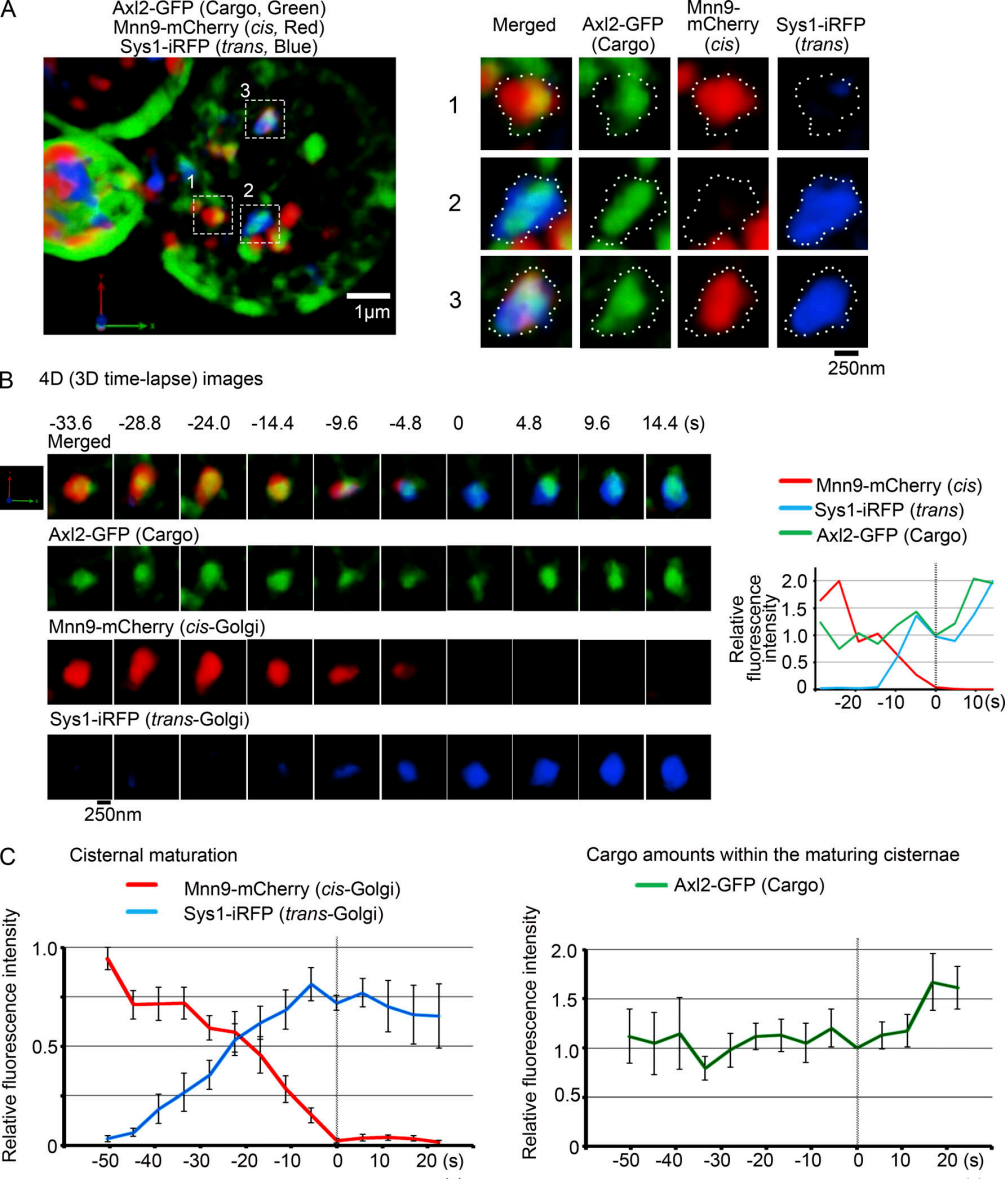
**Secretory cargo Axl2-GFP is transported from cis- to trans-Golgi while being maintained within a maturing cisterna.** *uso1-1* cells expressing heat-shock–inducible Axl2-GFP (cargo, green) and constitutive Mnn9-mCherry (cis-Golgi, red) and Sys1-iRFP (trans-Golgi, blue) were incubated at 37°C for 30 min and then shifted down to 24°C and observed by SCLIM. **(A)** A representative 3D image of cargo and Golgi markers is shown on the left. Axl2-GFP (cargo, green) was detected in the ER and the Golgi cisternae in the right cell and on the plasma membrane in the left cell. Scale bar, 1 µm. Right panels show enlarged 3D images of the selected cisternae with cargo in the boxed areas of the left panel. Merged and individual images of Axl2-GFP (cargo, green), Mnn9-mCherry (cis, red), and Sys1-iRFP (trans, blue) are shown. Scale bar, 250 nm. **(B)** A representative 4D (3D time-lapse) observation of a maturing cisterna. 3D images were reconstructed from 16 optical slices 200 nm apart around the center of the cell taken at 4.8-s intervals. The frame where Mnn9-mCherry signals disappeared was set as time 0. Merged (cargo, cis, and trans) and individual images are shown. Cargo was maintained within the Golgi cisterna, which matured from cis- to trans-Golgi. Scale bar, 250 nm. Relative fluorescence intensities of cargo, cis-Golgi, and trans-Golgi markers of the selected cisterna are shown on the right. **(C)** Quantification of Golgi markers and cargo during the cisternal maturation from cis- to trans-Golgi. Fluorescence intensities of cargo, cis-Golgi, and trans-Golgi markers of selected cisternae from nine independent maturation events were quantified with Z-stacks collected every 5.6 s. The frames where Mnn9-mCherry signals disappeared were set as time 0. For each of the nine maturing cisternae, the maximum fluorescence intensities of Mnn9-mCherry or Sys1-iRFP over time were set to 1 (left). For the trace of cargo, Axl2-GFP fluorescence intensity at time 0 in each of the nine experiments was normalized to 1 (right). Mean values of all the experiments per each time point were subjected to statistical analysis. Because of setting time 0 as the frames where Mnn9-mCherry signals disappeared, the number of maturing cisternae at each time point were five cisternae from −50.4 s to −44.8 s, seven cisternae at −39.2 s, eight cisternae from 33.6 s to 22.4 s, nine cisternae from −22.4 s to 0 s, eight cisternae at 5.6 s, seven cisternae at 11.2 s, and six cisternae from 16.8 s to 22.4 s. Bars represent standard errors of means.

We next conducted simultaneous visualization of secretory cargo delivery during the Golgi cisternal maturation. We have developed a fluorescent microscopic pulse-chase assay system to visualize a newly synthesized cargo tagged with a fluorescent protein in living yeast cells (Kurokawa et al., 2014). As a cargo we chose Axl2, a transmembrane protein targeted to the plasma membrane at the bud tip and the bud neck (Roemer et al., 1996). *uso1-1* temperature-sensitive mutant cells, which lack the Uso1 tethering function and block ER-to-Golgi cargo transport at the restrictive temperature (37°C; Nakajima et al., 1991; Lord et al., 2013), were transformed to express heat-shock–inducible Axl2-GFP (transmembrane cargo) and constitutive Mnn9-mCherry (cis-Golgi marker) and Sys1-iRFP (trans-Golgi marker). After preincubation at 37°C to synthesize and accumulate the cargo in the ER and then temperature shift down to 24°C, Axl2-GFP signals changed its location from the peripheral and tubular ER, to the Golgi cisternae, and finally to the bud (Fig. 4 A, left). Golgi cisternae with Axl2-GFP were colabeled with either Mnn9-mCherry or Sys1-iRFP depending on the stage of cargo transport (Fig. 4 A, cisternae in right panels). As shown in Fig. 4 B and Fig. S2, 3D time-lapse images of selected cisternae demonstrate that Mnn9-mCherry–positive cis-cisternae with Axl2-GFP matured into trans-Golgi cisternae labeled with Sys1-iRFP, while maintaining cargo (Videos 4 and 5). Mnn9-mCherry–positive cis-Golgi cisterna showed the hug-and-kiss action as observed previously (Kurokawa et al., 2014); cis-Golgi approached and contacted the ER at the ER exit sites, captured transmembrane cargo Axl2-GFP, and then left the ER together with cargo (Video 5). Once cis-Golgi cisternae acquired the cargo, they eventually matured into trans-Golgi cisternae. These results support that “cisternal maturation” of the Golgi is responsible for cargo transport in the Golgi apparatus. Fig. 4 C shows the result of statistic analysis on the fluorescence intensities of Mnn9-mCherry, Sys-iRFP, and Axl2-GFP over time from nine independent maturation events, clearly indicating maturation of cisternae from cis to trans while maintaining cargo. Note that the amount of transmembrane cargo appeared to increase slightly after the disappearance of Mnn9-mCherry.

**Figure 5.**
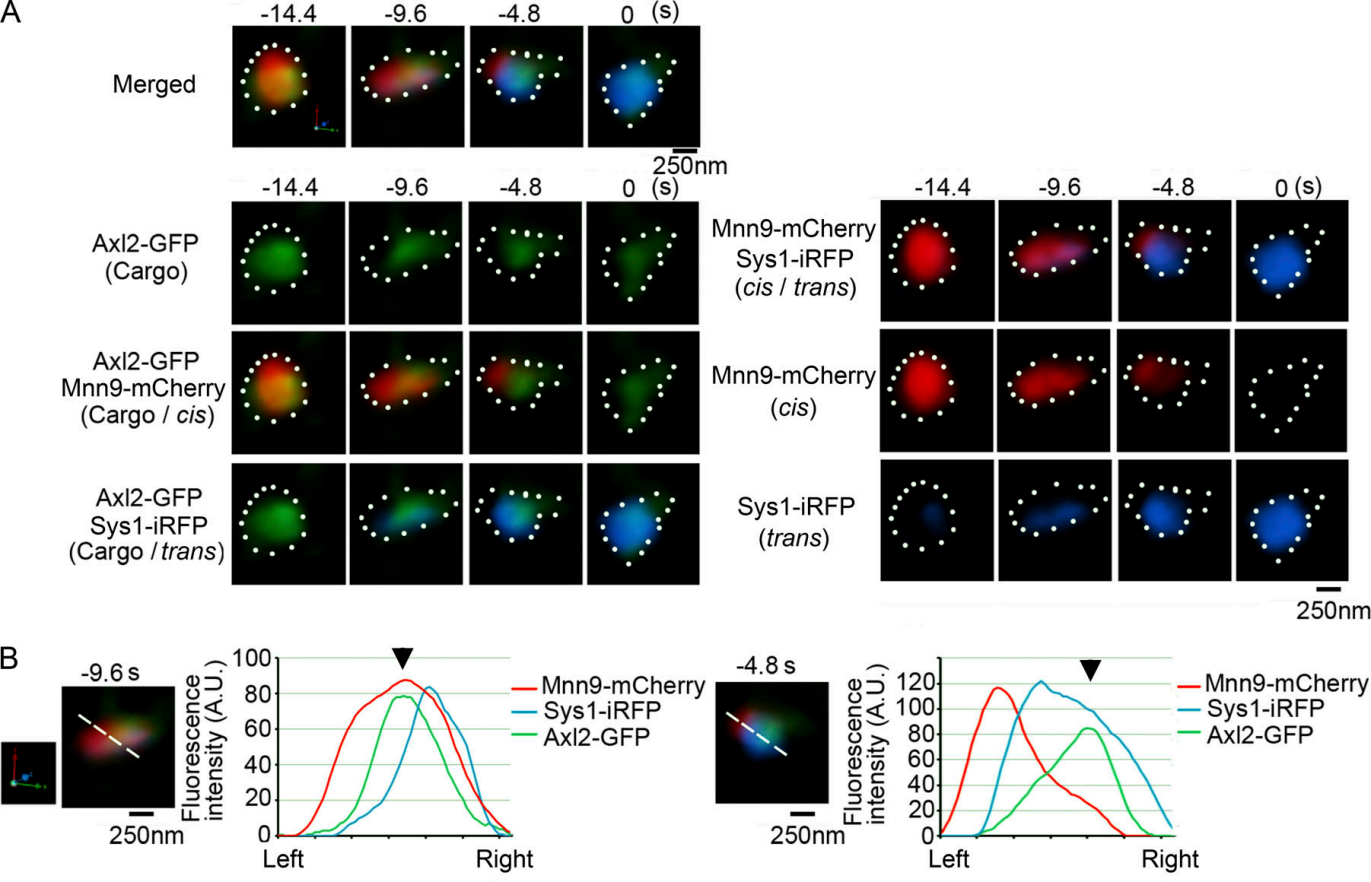
**Cargo moves from cis- to trans-Golgi zones within a cisterna during maturation. (A)** Top panels show enlarged images of Axl2-GFP (cargo, green), Mnn9-mCherry (cis-Golgi, red), and Sys1-iRFP (trans-Golgi, blue) during the transitional period of cisternal maturation (−14.4 to 0 s) shown in Fig. 4 B. The second to fourth rows in the left show cargo localization within the maturing cisterna, which harbors distinct zones labeled with cis- and trans-Golgi markers. Right panels show the segregation of cis- and trans-zones in the maturing cisterna. Scale bar, 250 nm. **(B)** Graphs show relative fluorescence intensities of Axl2-GFP (cargo, green), Mnn9-mCherry (cis, red), and Sys1-iRFP (trans, blue) along the white lines in the maturing cisterna at −9.6 s (left) and −4.8 s (right). Arrowheads indicate the peak positions of Axl2-GFP fluorescence intensities. Scale bar, 250 nm.

We next focused our interest on the intermediate state of the transition of cisternae from cis to trans. As demonstrated in Fig. 5 A, the trans marker Sys1-iRFP began to emerge as a small zone (−9.6 s) in the cis-Golgi cisterna labeled with Mnn9-mCherry, increased its volume without large mixing with the cis zone (−4.8 s), and finally concurred the entire region of the cisterna (0 s; see also Fig. 4 B). Notably, during the transitional period of cisternal change from cis to trans, the transmembrane cargo showed a nonuniform distribution in the cisterna (Fig. 5 A). As the trans-Golgi marker expanded the region, the cargo changed its location from the cis-Golgi zone to the trans-Golgi zone in the cisterna (Fig. 5 A, from −14.4 to −4.8 s). At −4.8 s, the two Golgi markers were segregated in the cisterna and the cargo appeared to have moved almost completely from the cis-Golgi zone to the newly formed trans-Golgi zone (Fig. 5 B, arrowheads). Movement of cargo and Golgi markers is a relative issue, and what looks like cargo movement could be due to leaving and arriving of Golgi markers on different sides. During the transitional period while Golgi cisternae matured from cis to trans (Fig. S2), the segregation of two Golgi markers was observed in eight of 11 maturing cisternae. The movement of cargo from the cis-Golgi zone to the trans-Golgi zone was also observed within all the maturing cisternae that showed the segregation of the two Golgi markers (8 of 11 cisternae; Fig. S3). These results indicate that during the cisternal maturation the cargo traverses different zones of Golgi residents.

### Secretory cargo is maintained within a cisterna but delivered in a dynamic way from cis-Golgi to the TGN

**Figure 6.**
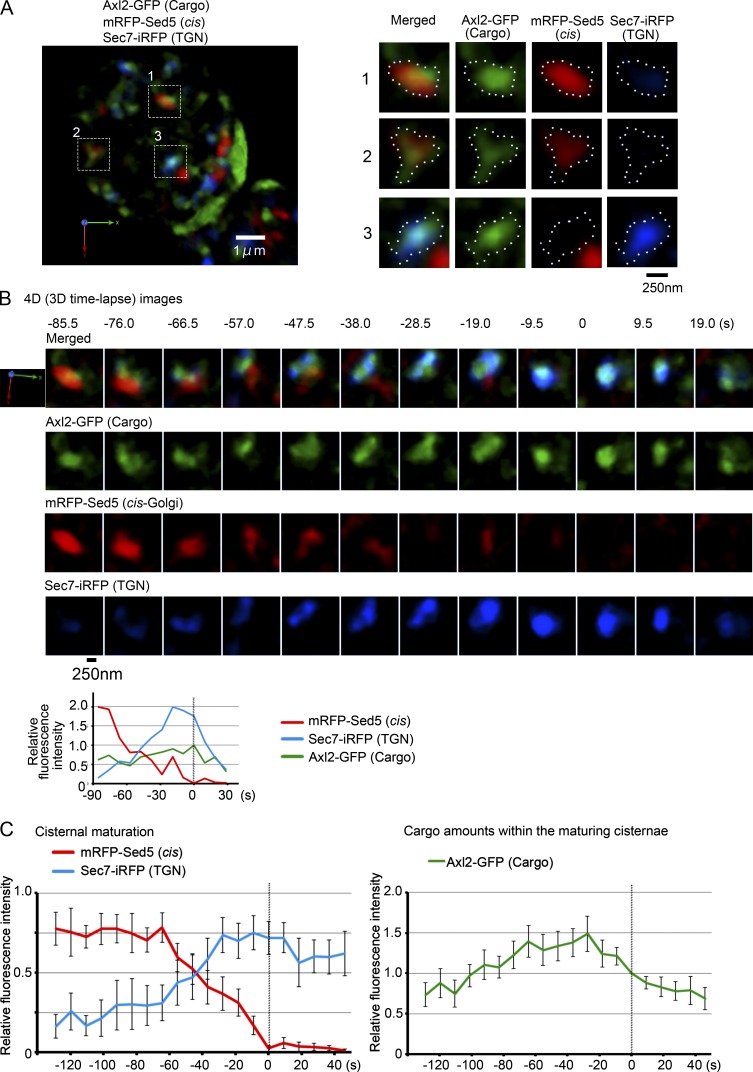
**Cargo is transported from cis-Golgi to the TGN while maintained in a maturing cisterna.** *uso1-1* cells expressing heat-shock–inducible Axl2-GFP (cargo, green) and constitutive mRFP-Sed5 (cis-Golgi, red) and Sec7-iRFP (TGN, blue) were incubated at 37°C for 15 min and then shifted down to 24°C and observed by SCLIM. **(A)** A representative 3D image is shown on the left. Axl2-GFP was detected on the Golgi cisternae and the plasma membrane. Scale bar, 1 µm. Right panels show enlarged 3D images of the selected cisternae shown in boxes in A. Merged and individual images of Axl2-GFP (cargo, green), mRFP-Sed5 (cis, red), and Sec7-iRFP (TGN, blue) are shown. Scale bar, 250 nm. **(B)** 4D observation of cisterna #1 of A. 3D images were reconstructed from 31 optical slices 200 nm apart taken at 9.5-s intervals. Merged (cargo, cis, and TGN) and individual images are shown. Scale bar, 250 nm. Relative fluorescence intensities of cargo, cis-Golgi, and TGN markers in the cisterna are shown in the lower graph. **(C)** Quantification of Golgi/TGN markers and cargo Axl2 during the cisternal maturation from cis-Golgi to the TGN. Fluorescence intensities of cargo, cis-Golgi, and TGN markers of selected cisternae from 17 independent maturation events were quantified with Z-stacks collected every 9.2 s. The frames where mRFP-Sed5 signals disappeared were set as time 0. For each of the 17 maturing cisternae, the maximum fluorescence intensities of mRFP-Sed5 or Sec7-iRFP over time was set to 1 (left). For the trace of cargo, Axl2-GFP fluorescence intensity at time 0 in each of the 17 experiments was normalized to 1 (right). Mean values of all the experiments per each time point were subjected to statistical analysis. Because of setting time 0 as the frames where mRFP-Sed5 signals disappeared, the number of maturing cisternae at each time point were 6 cisternae from −128.8 to −119.6 s, 8 cisternae at −110.4 s, 11 cisternae at 101.2 s, 12 cisternae at −92.0 s, 13 cisternae at −82.8 s, 15 cisternae from −73.6 to −55.2 s, 16 cisternae from −46.0 to −36.8 s, 17 cisternae from −27.6 to 0 s, 16 cisternae at 9.2 s, 15 cisternae at 18.4 s, 13 cisternae at 27.6 s, 12 cisternae at 36.8 s, and 11 cisternae at 46.0 s. Error bars represent standard errors of means.

To further verify that the cargo transport is achieved by relocation through the zones formed in a cisterna, we next observed cargo behaviors over a longer period from cis-Golgi to the TGN. mRFP-Sed5 and Sec7-iRFP were observed, which show overlapping residence on a maturing cisterna during this period. We conducted three-color 4D SCLIM observation of the *uso1-1* cells expressing heat-shock–inducible Axl2-GFP (cargo) and mRFP-Sed5 (cis-Golgi marker) and Sec7-iRFP (TGN marker). Upon the temperature shift down to the permissive temperature, the cargo Axl2-GFP signal was observed in the Golgi cisternae labeled with cis-Golgi and TGN markers (Fig. 6 A). 3D time-lapse images of one cisterna harboring Axl2-GFP (Fig. 6, A [square #1] and B; and Fig. S4) demonstrated that the cis-Golgi cisterna labeled with mRFP-Sed5 matured to the TGN labeled with Sec7-iRFP while maintaining cargo (see also Video 6). Fig. 6 C shows the result of statistic analysis on the fluorescence intensities of mRFP-Sed5, Sec7-iRFP, and Axl2-GFP over time from 17 independent maturation events. The amount of transmembrane cargo showed transient increase before the complete disappearance of mRFP-Sed5 (time 0) and then decreased during colocalization with the Sec7-iRFP signal (Fig. 6 C). This result together with that of Fig. 4 C raises a possibility that the cargo might be transported from elsewhere during the transition period from trans-Golgi to TGN.

**Figure 7.**
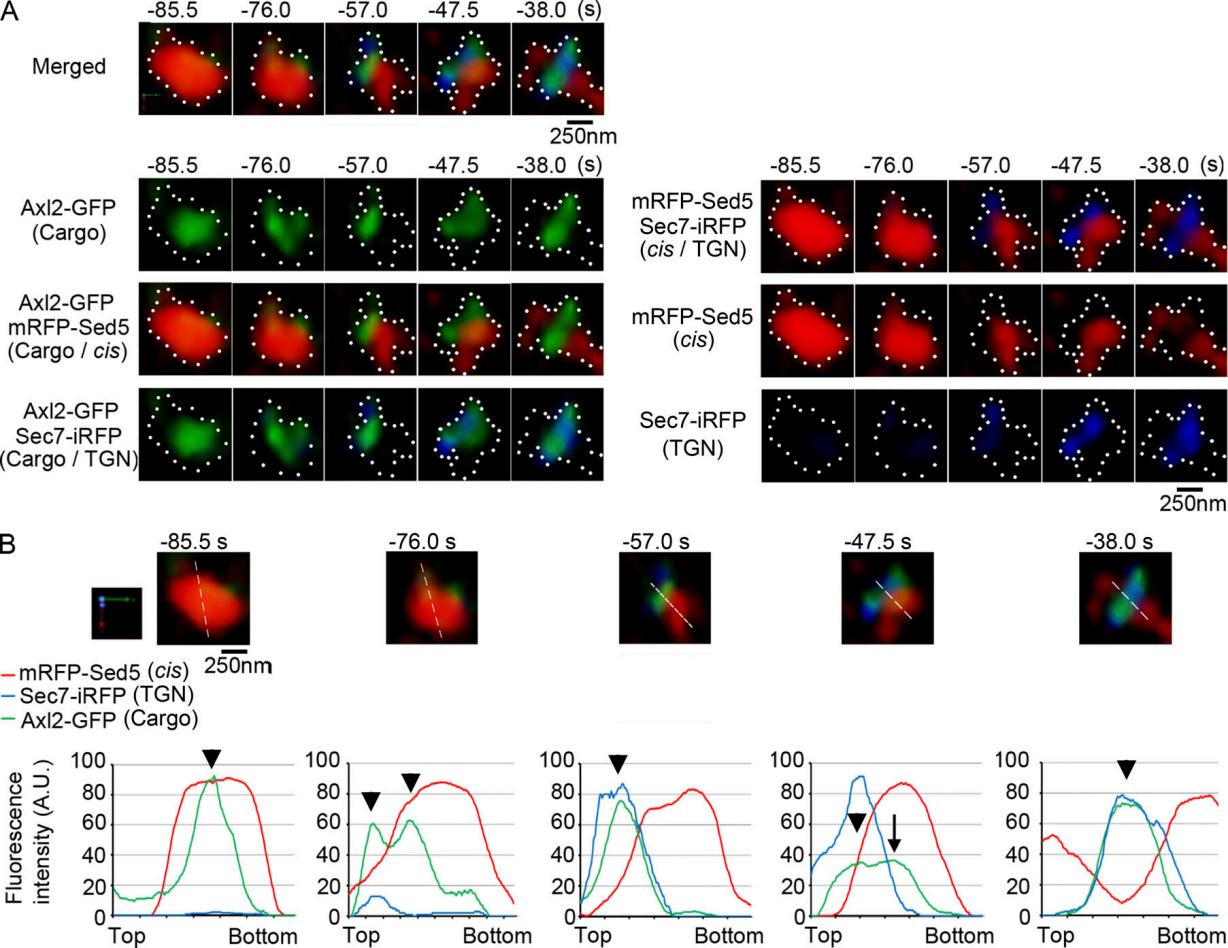
**Cargo sometimes shows a peculiar behavior between the zones within a maturing cisterna. (A)** Top panels show enlarged images of cargo (green), cis-Golgi (red), and TGN (blue) markers during the transitional period of cisternal maturation (−85.5 to −38.0 s) in Fig. 6 B. The second to fourth rows in the left show cargo localization within the maturing cisterna. Note the presence of distinct zones labeled with cis-Golgi and TGN markers. Right panels show the transition from cis to TGN zones during maturation, which manifests clear segregation. Scale bar, 250 nm. **(B)** Graphs show relative fluorescence profiles of cargo (green), cis-Golgi (red), and TGN (blue) markers along the white lines in the maturing cisterna shown above. Note that cargo almost reached the TGN zone at −57.0 s (see arrowheads), but a small peak reappeared in the cis-Golgi zone at −47.5 s (arrow at −47.5 s). Cargo finally accumulated in the TGN zone (arrowhead at −36.0 s). Arrowheads indicate the peak positions of Axl2-GFP fluorescence intensities. Scale bar, 250 nm.

We noticed again that earlier and later Golgi markers displayed segregation in the maturing cisterna. The cargo Axl2-GFP did not show uniform distribution in the cisterna but exhibited biased localization (Figs. 6 B and 7). Sec7-iRFP first emerged in a small zone on the cis-Golgi cisterna labeled with mRFP-Sed5 and then expanded its area (Figs. 6 B and 7 A, −85.5 to −76.0 s). During the transition phase of the cisterna changing its nature from cis-Golgi to the TGN, clear segregation was observed in the zones of two different Golgi markers (Figs. 6 B and 7 A, −76.0 to −47.5 s). The cargo began to enter a small zone labeled with the TGN marker (Fig. 7, A and B, −85.5 to −76.0 s, left arrowhead at 76.0 s in B) and eventually relocated to the expanding TGN zone (Fig. 7, A and B, −76.0 to −57.0 s, arrowhead at −57.0 s in B). The segregation of early and late Golgi markers and the movement of transmembrane cargo between their zones were observed in 14 of 19 maturing cisternae (Fig. S5). Interestingly, some portion of cargo showed a peculiar behavior, which looked like backward relocation. After almost the complete shift to the TGN zone, a small peak of cargo reappeared in the cis-Golgi–labeled zone (Fig. 7 B, −57.0 to −47.5 s, arrow at −47.5 s; and Fig. S5, arrows). These behaviors were observed in eight of the 14 cisternae that showed clear segregation of early and late markers. Cargo reaching the TGN zone finally occupied there (Fig. 7 B, −38.0 s, arrowhead; and Fig. S5, right panels and graphs).

**Figure 8.**
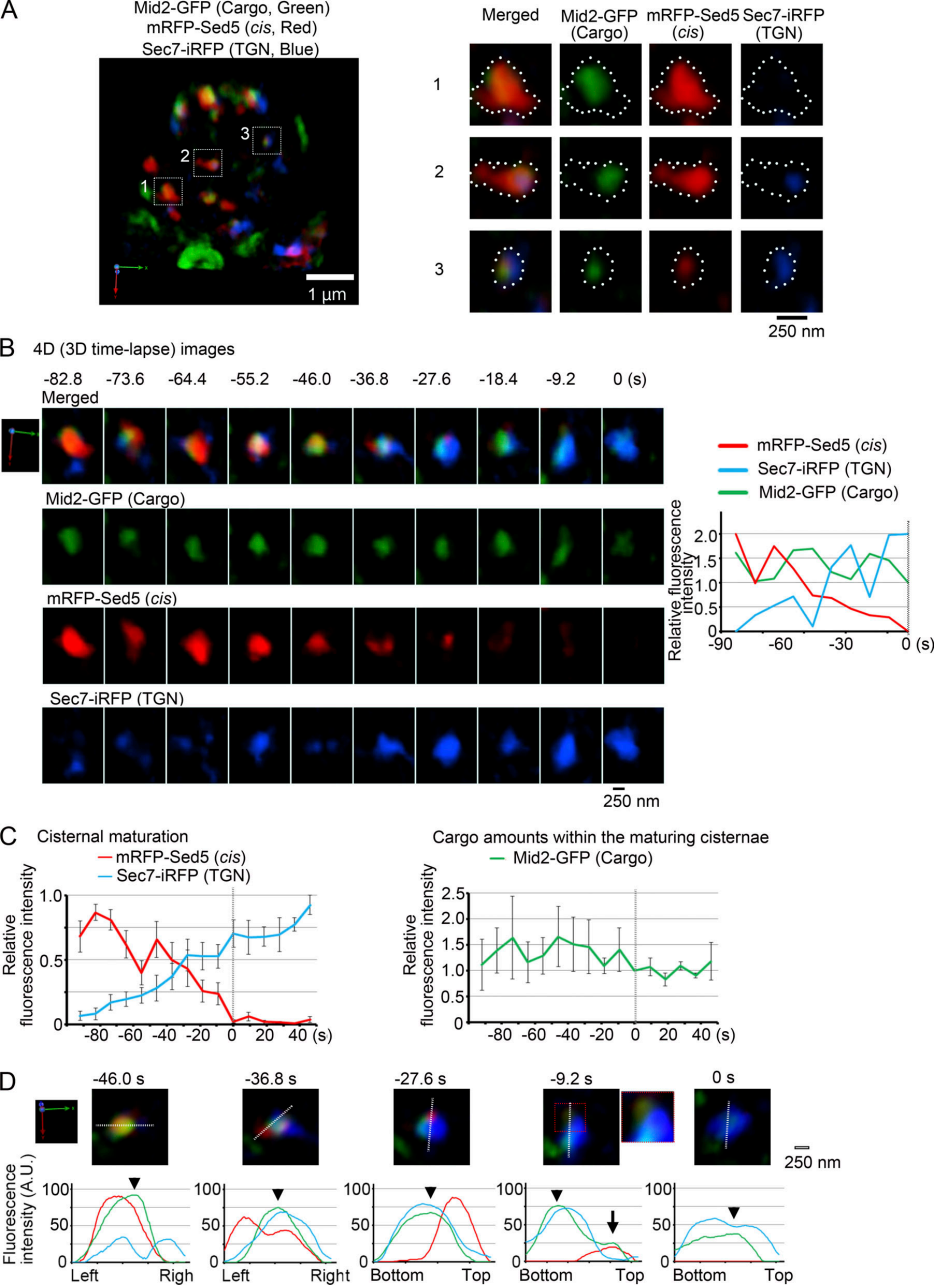
**Another transmembrane cargo Mid2-GFP is also transported from cis-Golgi to the TGN while maintained in a maturing cisterna.** *uso1-1* cells expressing heat-shock–inducible Mid2-GFP (cargo, green) and constitutive mRFP-Sed5 (cis-Golgi, red) and Sec7-iRFP (TGN, blue) were incubated at 37°C for 15 min and then shifted down to 24°C and observed by SCLIM. **(A)** A representative 3D image is shown on the left. Mid2-GFP was detected on the Golgi cisternae and the plasma membrane. Scale bar, 1 µm. Right panels show enlarged 3D images of the selected cisternae shown in boxes in A. Merged and individual images of Mid2-GFP (cargo, green), mRFP-Sed5 (cis, red), and Sec7-iRFP (TGN, blue) are shown. Scale bar, 250 nm. **(B)** Representative 4D observation. 3D images were reconstructed from 26 optical slices 200 nm apart taken at 9.2-s intervals. Merged (cargo, cis, and TGN) and individual images are shown. Scale bar, 250 nm. Relative fluorescence intensities of cargo, cis-Golgi, and TGN markers in the cisterna are shown on the right. **(C)** Quantification of Golgi/TGN markers and cargo Mid2 during the cisternal maturation from cis-Golgi to the TGN. Fluorescence intensities of cargo, cis-Golgi, and TGN markers of selected cisternae from seven independent maturation events were quantified with Z-stacks collected every 9.2 s. The frames where mRFP-Sed5 signals disappeared were set as time 0. For each of the seven maturing cisternae, the maximum fluorescence intensities of mRFP-Sed5 or Sec7-iRFP over time was set to 1 (left). For the trace of cargo, Mid2-GFP fluorescence intensity at time 0 in each of the seven experiments was normalized to 1 (right). Mean values of all the experiments per each time point were subjected to statistical analysis. Because of setting time 0 as the frames where mRFP-Sed5 signals disappeared, the number of maturing cisternae at each time point were five cisternae at −92.0 s, six cisternae at −82.8 to −46.0 s, seven cisternae from −36.8 to 0 s, five cisternae from 9.2 to 27.6 s, and four cisternae from 36.8 to 46.0 s. Error bars represent standard errors of means. **(D)** Top panels show merged images of cargo (green), cis-Golgi (red), and TGN (blue) markers during the transitional period of cisternal maturation (−46.0 to 0 s) in B. Graphs show relative fluorescence profiles of cargo (green), cis-Golgi (red), and TGN (blue) markers along the white lines in the cisterna shown above. Cargo moved from the cis-Golgi zone to the TGN zone completely (−46.0 to −27.6 s, see arrowheads). Note that a small peak of cargo reappeared in the cis-Golgi zone (arrow at −9.2 s) but disappeared and merged again with TGN (0 s). An enlarged image of the red dotted region at −9.2 s is shown on the right. Arrowheads indicate the peak positions of Axl2-GFP fluorescence intensities.

To validate the generality of such dynamic cargo behaviors, we next looked at another transmembrane cargo Mid2-GFP to see how it behaves during cisternal maturation from cis-Golgi to the TGN. Mid2 is a sensor for cell wall integrity signaling, and its final destination is the plasma membrane (Ono et al., 1994). We conducted three-color 4D SCLIM observation of the *uso1-1* cells expressing heat-shock–inducible Mid2-GFP (cargo) and mRFP-Sed5 (cis-Golgi marker) and Sec7-iRFP (TGN marker). Upon the temperature shift down to the permissive temperature, the cargo Mid2-GFP signals were observed in the Golgi cisternae labeled with early and late markers (Fig. 8 A). 3D time-lapse images of a cisterna harboring Mid2-GFP exhibited maturation from the cis-Golgi cisterna to the TGN while maintaining cargo (Fig. 8 B). The result of quantification and statistic analysis of these three fluorescence images is shown in Fig. 8 C. Earlier and later Golgi markers displayed segregation in the maturing cisterna, and the transmembrane cargo moved from the early Golgi zone to the later zone (Fig. 8, B and D, −46.0 to −27.6 s, see arrowheads in D). The segregation of different Golgi markers and the movement of cargo between the zones were observed in five of seven cisternae. The reappearance of cargo in the earlier zone was also observed (Fig. 8 D, −9.2 s, arrow) in four of the five cisternae with clear segregation of early and late markers. These results indicate again that transmembrane cargo stays in the cisterna during maturation from cis-Golgi to the TGN, while a small portion of cargo sometimes shows apparent and transient backward relocation in the earlier zone.

### COPI function is required for completion of maturation and delivery of cargo to further destinations

**Figure 9.**
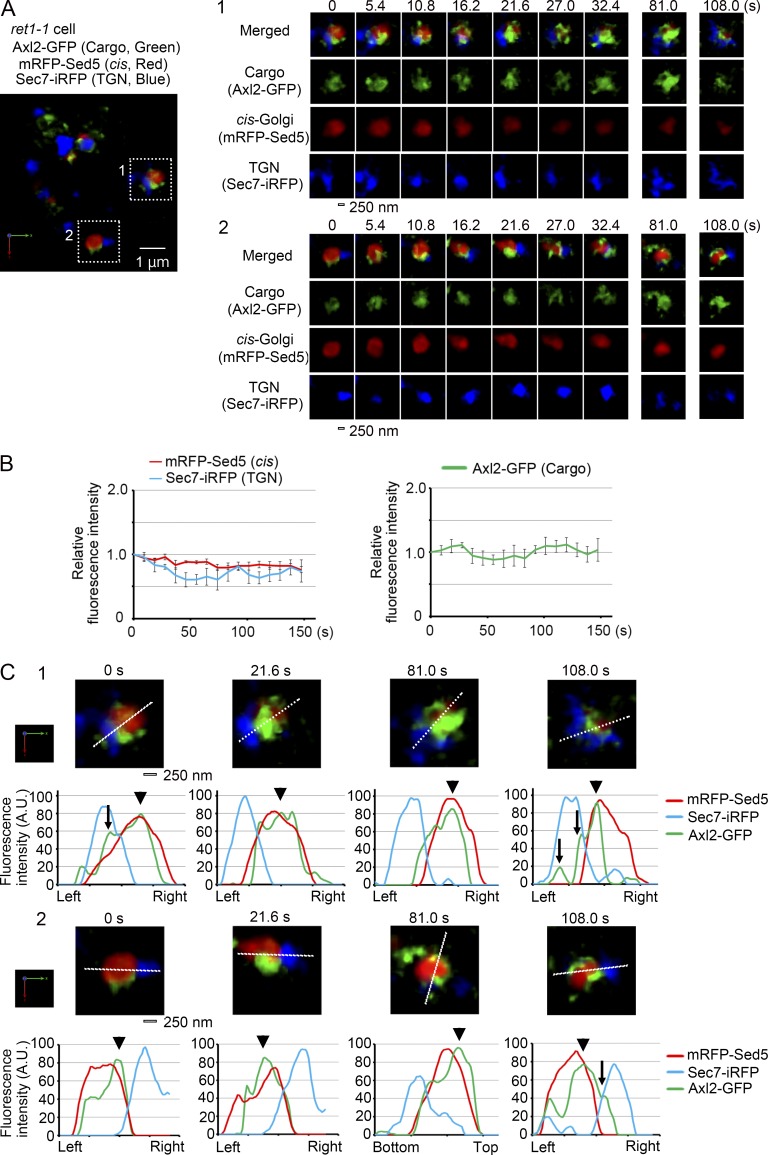
**Cargo transfer from the early to the late zone is prevented in the cells deficient in COPI function.** *ret1-1* cells expressing heat-shock–inducible Axl2-GFP (cargo, green) and constitutive mRFP-Sed5 (cis-Golgi, red) and Sec7-iRFP (TGN, blue) were incubated at 37°C for 30 min and then observed on the stage at 37°C by SCLIM. **(A)** A representative 3D image is shown on the left. Axl2-GFP was detected on the Golgi cisternae labeled with mRFP-Sed5 and Sec7-iRFP. Scale bar, 1 µm. Right panels show the time-lapse 3D images of the selected cisternae shown in boxes in A. 3D images were reconstructed from 16 optical slices 200 nm apart taken at 5.4-s intervals. Merged (cargo, cis, and TGN) and individual images are shown. Scale bar, 250 nm. **(B)** Quantification of Golgi/TGN markers and cargo Axl2 during the observation period. Fluorescence intensities of cargo, cis-Golgi, and TGN markers of five selected cisternae were quantified with Z-stacks collected every 9.2 s. The fluorescence intensities of mRFP-Sed5, Sec7-iRFP, or Axl2-GFP at time 0 in each of the five experiments were set to 1. Mean values of all the experiments per each time point were subjected to statistical analysis. Bars represent standard errors of means. **(C)** The panels in the first and the third rows show enlarged merged images of cargo (green), cis-Golgi (red), and TGN (blue) markers at indicated times of cisternae #1 and #2 of A, respectively. The graphs of the second and fourth rows show relative fluorescence profiles of cargo (green), cis-Golgi (red), and TGN (blue) markers along the white lines in the cisterna shown above. Note that most of cargo stayed in the cis-Golgi zone (arrowheads) and a small portion of cargo appeared in the TGN zone occasionally and transiently (arrows). Scale bar, 250 nm.

We have previously shown that the function of COPI is essential for Golgi cisternal maturation and dynamics (Ishii et al., 2016). To obtain a hint to understand the mechanism of cargo relocation within the maturing Golgi cisterna, we examined cargo behavior in the COPI α-subunit temperature-sensitive mutant *ret1-1*, which is defective in COPI vesicle formation at the restrictive temperature. We conducted three-color 4D SCLIM observation of the *ret1-1* cells expressing heat-shock–inducible Axl2-GFP (cargo) and mRFP-Sed5 (cis-Golgi marker) and Sec7-iRFP (TGN marker) at the restrictive temperature. We could find cells in which the cargo Axl2-GFP signal was present in the Golgi cisterna labeled with both mRFP-Sed5 and Sec7-iRFP (Fig. 9 A). 3D time-lapse images of these Axl2-GFP-harboring cisternae showed that cargo was maintained in the cisterna in which mRFP-Sed5 and Sec7-iRFP stayed persistently segregated (Fig. 9 A). Fig. 9 B shows the result of statistical analysis on the fluorescence intensities of mRFP-Sed5, Sec7-iRFP, and Axl2-GFP over time from five independent cisternae, clearly indicating the defect of cisternal maturation in *ret1-1* cells at the restrictive temperature. Most cargo stayed overlapping with the cis-Golgi marker (Fig. 9 C, arrowheads), while a small portion of cargo was found in the TGN zone occasionally and transiently (Fig. 9 C, arrows). In all seven independent cisternae observed, cargo was never able to leave the cis-Golgi zone completely. These results indicate that efficient delivery of cargo from the cis-Golgi to subsequent destinations requires COPI.

## Discussion

Live imaging of yeast Golgi provided strong evidence to support the cisternal maturation model (Glick and Nakano, 2009). Cisternae labeled with different fluorescent proteins changed colors over time in the cis-to-trans direction (Losev et al., 2006; Matsuura-Tokita et al., 2006). However, an important piece of evidence was lacking at that time, namely, visualization of secretory cargo, which was supposed to stay in the maturing cisternae. Tracking of cargo is not easy, because it goes through the secretory pathway in a constitutive flow at a considerable speed. To observe cargo movement from one compartment to another, pulse-chase experiments are required, in which visible cargo is made in a short period of time and then gets transported.

We established such a pulse-chase imaging system, taking advantage of yeast genetics (Kurokawa et al., 2014). Transmembrane secretory cargo Axl2-GFP was put under control of the HSP70 heat-shock promoter and expressed in temperature-sensitive secretory mutant cells, such as *uso1-1* and *sec31-1*. When the cells are incubated at high temperature, cargo Axl2-GFP is synthesized but remains in the ER due to the secretory block. Upon shift down to low temperature, new synthesis of cargo is shut down and the already-synthesized Axl2-GFP starts trafficking from the ER as the secretory block is released.

In the present study, we applied this pulse-chase imaging to follow the behavior of cargo during cisternal maturation of the Golgi. By three-color and 4D SCLIM observation, we could indeed demonstrate that cargo remains in the cisterna that is changing its property from cis to trans and to TGN during maturation (Figs. 4, 5, 6, 7, and 8). Interestingly, cargo does not occupy the whole area of the maturing cisterna, but is rather in a restricted zone. During the period that two distinct markers such as cis and trans or cis and TGN are present in a single cisterna in a segregated way, cargo changes its location from the cis-zone to the trans-zone and to the TGN zone (Figs. 4, 5, 6, 7, 8, S3, and S5). In our previous studies (Matsuura-Tokita et al., 2006; Ishii et al., 2016), we realized that during maturation, dynamic mixing and segregation of different Golgi resident proteins occurred; in other words, distinct zones were present in the maturing cisternae. The present study clearly shows that cargo relocates from the early to late zones in such a dynamic process of the maturing cisterna. We also show that this transfer of cargo from the early to the late zone requires the COPI function (Fig. 9). COPI is thought to sequester Golgi resident proteins in a retrograde way, leaving cargo in the cisterna. Thus disappearance of earlier residents and emergence of later residents, both in a COPI-dependent fashion, is probably one of the major mechanisms of apparent transfer of cargo from the earlier to the later zone within the maturing Golgi cisterna.

A caveat of fluorescence imaging is that only the regions where fluorescent proteins are present can be visualized. The SCLIM imaging has achieved a spatial resolution beyond the diffraction limit, but even so a question remains whether the zones present in what looks like a maturing cisterna are indeed in a continuous membrane entity. To address this problem, we conducted a live CLEM analysis combining 4D SCLIM with TEM observation. After appropriate correlation of the data obtained by light and electron microscopy, the 3D-reconstructed images corresponded very well to each other, indicating that our fluorescence imaging is visualizing almost the whole structures of the maturing cisternae (Fig. 3). The 3D images of the cisterna in transition from cis to TGN reconstructed by SCLIM and TEM (Fig. 3 F) both look fenestrated, like tubular network structures as elaborated in previous EM studies (Rambourg et al., 2001; Beznoussenko et al., 2016).

During the course of 4D SCLIM analysis of cargo delivery, we found a very curious behavior of cargo. In the case of maturation from cis-Golgi to the TGN, after cargo almost reached the TGN zone, a small amount of cargo often reappeared in the cis zone (Figs. 6, 7, 8, S4, and S5). This looks as if a part of cargo relocated backward from the TGN to the cis-Golgi zone. If cargo is to be kept still in a cisterna in a static fashion as proposed in the classic cisternal maturation model, this phenomenon needs an explanation. One intriguing idea might be that a kind of quality control system exists in the Golgi, and cargo that has not completed adequate modifications in the earlier zone is sent back from the later to the earlier zone. We notice there is a variety in the rate of cisternal maturation even in a single cell. Cargo delivery from earlier to later compartments in the Golgi may not be a simple static process but involves dynamic, stochastic, and perhaps fail-safe mechanisms. A mechanism might exist to check the cargo status, whether it is ready to go to the next step of transport. Alternatively, what looked like a backward relocation could be due to another influx of cargo into the earlier zone during the maturation from the cis-Golgi to the TGN. Interestingly, the amount of cargo in the maturing cisterna appeared to show a slight increase in trans-Golgi during the cis-to-trans maturation (Fig. 4 C) and a transient increase and decrease during the maturation from cis-Golgi to the TGN (Fig. 6 C), suggesting a possibility that cargo may be transported from other compartments at the boundary between trans-Golgi and the TGN. This may imply a special function of the TGN, perhaps linking its roles for both the exocytic and endocytic pathways (Day et al., 2018; unpublished data). Anyway, if such an additional entry of cargo into a maturing cisterna were the case, what mechanism could bring it would be a new question.

As for the basic mechanisms of cargo delivery within the Golgi, our findings support the cisternal maturation to be the main backbone. However, we should also appreciate that the behavior of cargo is very dynamic even in a maturing cisterna. Passive residence in the mixing cisternae is probably not sufficient to explain dynamic behavior of cargo, mixing with and segregating from Golgi resident proteins. In that sense, some other models postulating compartmentalization within cisternae may be worth revisiting (Patterson et al., 2008; Pfeffer, 2010).

Our data are also compatible with the idea that the interconnected continuity of cisternae plays a role in trafficking (Trucco et al., 2004; Pfeffer, 2010; Beznoussenko et al., 2014). Unstacked Golgi cisternae of yeast frequently show dynamic features of contact and separation, which could be considered as temporal continuity between cisternae. Interconnection between different Golgi cisternae has also been reported by tomographic observation of yeast (Beznoussenko et al., 2016). Because the cargo molecules (Axl2 and Mid2) we are looking at in the present study are transmembrane proteins, simple diffusion is unlikely. How the directionality of cargo delivery is ensured in the complex structures of cisternae has to be addressed.

We have previously shown that COPI is essential for cisternal maturation (Ishii et al., 2016). In the present article, we show that COPI function is indeed required for efficient delivery of cargo from the cis-Golgi zone to further destinations. Sometimes small punctate structures are seen in our 4D imaging, but whether they are COPI coated remains to be examined. Sec7 mainly marks the TGN, because the Sec7 compartment colocalizes well with clathrin and clathrin adaptor molecules but not with COPI (unpublished data). Nevertheless, cisternal maturation to the Sec7 TGN compartment is also severely impaired by inactivation of COPI (Fig. 9; see also Ishii et al., 2016). The mechanism of transition from the trans-cisterna of the Golgi to the TGN needs further investigations.

The involvement of anterograde vesicular or tubular carriers is not ruled out by the live imaging we performed in the present study. A recent report from Rothman’s group implicated evidence for anterograde carriers between the mammalian Golgi cisternae separated by locking on mitochondria (Dunlop et al., 2017). Live imaging at higher speed and at better spatial resolution is earnestly awaited. SCLIM2, the next-generation SCLIM we have recently developed, has achieved much improved spatiotemporal resolution that enables 4D tracking of vesicles in the cytoplasm (unpublished results). We are enthusiastic to tackle the many remaining questions with this new technology soon.

## Materials and methods

### Yeast strains, plasmids, and culture conditions

Yeast strains expressing Mnn9-mCherry and Sys1-2xGFP were constructed by a PCR-based method using pFA6a plasmids as a template (Huh et al., 2003; Suzuki et al., 2013) and primers 5′-ATT​TGG​CTT​ACC​AAA​CTA​TTT​GGT​TTA​TCA​CAT​AGA​GGA​AGA​GAA​CCA​TCG​GAT​CCC​CGG​GTT​AAT​TAA-3′ and 5′-ATT​ATC​TTT​CAA​TAA​CGC​TAT​AGC​TTC​TGT​ATG​CTT​TTT​GCT​CAG​TTG​CGA​ATT​CGA​GCT​CGT​TTA​AAC-3′ for Mnn9 and 5′-CAG​AAT​TGG​AGC​AAT​CAC​CAA​TAC​AAC​TAA​AAG​ACT​TAG​AAA​GCC​AAA​TA GGA​TCG​AGC​GCA​TCG​GGT​GC-3′ and 5′-ATG​ACT​ATA​ATA​TTG​CCA​AAA​TTG​CCT​CGA​ATA​TGT​TGT​TTC​ATC​TCC​TCATC​GAT​GAA​TTC​GAG​CTC​G-3′ for Sys1. mRFP-Sed5 was expressed under the control of the *TDH3* promoter on the low-copy plasmid pRS316 (Sato et al., 2001; Matsuura-Tokita et al., 2006). Sec7-GFP, Sec7-iRFP, and Sys1-iRFP were expressed similarly except that *ADH1* promoter was used instead of the *TDH3* promoter. Axl2-GFP and Mid2-GFP was expressed under the control of a heat shock promoter (*SSA1*) on the integration vector pRS304 (Kurokawa et al., 2014).

### Live-cell imaging by SCLIM

Yeast cells were grown in MCD medium (0.67% yeast nitrogen base without amino acids [Difco Laboratories], 0.5% casamino acids [Difco Laboratories], and 2% glucose) with appropriate supplements. For live imaging, cells were grown to a mid-log phase at 24°C. For pulse-chase imaging of Axl2-GFP and Mid2-GFP delivery, cells were chosen in which Axl2-GFP was properly delivered to the bud sites and Mid2-GFP was transported to the plasma membrane, because overexpression of Axl2 and Mid2 sometimes affects cell growth and accordingly its normal transport. A thermo-controlled stage (Tokai Hit) was used to observe *ret1-1* cells at the restrictive temperature. Cells were immobilized on glass slides using concanavalin A and imaged by SCLIM. SCLIM was developed by combining Olympus model IX-71 inverted fluorescence microscope with a UPlanSApo 100× NA 1.4 oil objective lens (Olympus), a high-speed and high signal-to-noise ratio spinning-disk confocal scanner (Yokogawa Electric), a custom-made spectroscopic unit, image intensifiers (Hamamatsu Photonics) equipped with a custom-made cooling system, magnification lens system for giving 266.7× final magnification, and three EM-CCD cameras (Hamamatsu Photonics) for green, red, and infrared observation (Kurokawa et al., 2013). Image acquisition was executed by custom-made software (Yokogawa Electric).

For 3D and 4D live imaging, we collected optical sections spaced 100 or 200 nm apart in stacks by oscillating the objective lens vertically with a custom-made piezo actuator. Z stack images were converted to 3D voxel data and processed by deconvolution with Volocity (Perkin Elmer) using the theoretical point-spread function for spinning-disk confocal microscopy. MetaMorph software (Molecular Devices) was used for presenting time-lapse images, fluorescence signal change analysis, and line-scan analysis.

### Live CLEM analysis

Live CLEM analysis was performed based on the live CLEM method for yeast cells described previously (Asakawa et al., 2010, 2014). We first conducted 4D live imaging of cells expressing Golgi markers by using SCLIM as described above except that optical sections were taken at 100 or 80 nm apart for this purpose. In the course of 4D imaging by SCLIM, the cells were fixed with glutaraldehyde by replacing the culture medium with 2% glutaraldehyde solution dissolved in phosphate buffer. After another few takes of 4D imaging by SCLIM, the cells were replenished with fresh 2% glutaraldehyde solution and incubated for 2 h at room temperature. After confirmation that Golgi markers no longer moved, bright-field and vacuolar intrinsic fluorescence 3D images of the fixed cells were taken as reference to be correlated to TEM images in the later analysis. The cells were then postfixed with 1.2% KMnO_4_ overnight, dehydrated, and embedded in epoxy resin. Thin sections (80 nm) of specimen were cut from the resin block using an ultramicrotome (Leica Biosystems) with a diamond knife. Serial sections were collected onto Formvar-coated copper slit grids, stained with uranyl acetate and lead citrate, and then analyzed by transmission electron microscope (JEM-1400; JEOL). For correlation, images obtained by SCLIM and TEM were compared and merged by adjusting cell shape and vacuolar shape using Microsoft PowerPoint. The cell sizes observed by TEM were 70–80% (dependent on individual cells and methods) compared with that observed by SCLIM. Therefore, 80-nm thickness of specimen for TEM analysis corresponded to about ∼100 nm in live cells, which is about same as the z-axis steps of 4D SCLIM observation (optical sections spaced 100 nm apart for the cell expressing Mnn9-mCherry and Sys1-2xGFP and 80 nm apart for the cell expressing mRFP-Sed5 and Sec7-GFP). Membrane images of the Golgi cisternae were traced, and 3D tomographic images were reconstructed by using Imaging Pro Premier 3D Software (Media Cybernetics).

### Online supplemental material

Fig. S1 shows the vacuolar membrane structure observed by SCLIM and TEM. Fig. S2 shows that secretory cargo is transported from cis- to trans-Golgi while being maintained within a maturing cisterna. Fig. S3 shows that cargo moves from cis- to trans-Golgi zones within a cisterna during maturation. Fig. S4 shows that cargo is transported from cis-Golgi to the TGN while being maintained in a maturing cisterna. Fig. S5 shows that cargo moves from cis-Golgi to the TGN within a cisterna during maturation. Video 1 is a three-color 4D observation of a WT cell expressing Mnn9-mCherry (cis-Golgi, red), Sys1-2xGFP (trans-Golgi, green), and Sec7-iRFP (TGN, blue). Video 2 is a multi-angle 3D reconstructed movie of a maturing cisterna (from cis- to trans-Golgi) and a cis-Golgi cisterna by SCLIM and TEM. Video 3 is a multi-angle 3D reconstructed movie of a maturing cisterna (from cis-Golgi to the TGN) by SCLIM and TEM. Video 4 is a three-color 4D movie of a Mnn9-mCherry–positive cis-cisterna (red) with cargo Axl2-GFP (green) that matures into trans-Golgi cisterna labeled with Sys1-iRFP (blue). Video 5 is another example of a three-color 4D movie of a Mnn9-mCherry–positive cis-cisterna (red) with cargo Axl2-GFP (green) that matures into trans-Golgi cisterna labeled with Sys1-iRFP (blue). Video 6 is a three-color 4D movie of cis-Golgi cisterna labeled with Sed5-mRFP (red) with cargo Axl2-GFP (green) maturing to the TGN labeled with Sec7-iRFP (blue).

## Supplementary Material

Supplemental Materials (PDF)

Video 1

Video 2

Video 3

Video 4

Video 5

Video 6
