# Supplementary material for: Visualization of secretory cargo transport within the Golgi apparatus
Source: J Cell Biol. 2019 Mar 11;218(5):1602–18. doi: 10.1083/jcb.201807194 (PMC6504898; doi:10.1083/jcb.201807194)
Supplement: Supplemental Materials (PDF) [file JCB_201807194_sm.pdf]

Supplemental material

Kurokawa et al., https://doi.org/10.1083/jcb.201807194

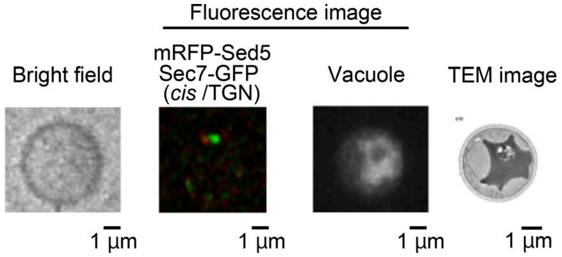

Figure S1. **Vacuolar membrane structure observed by SCLIM and TEM.** After 4D observation of cisternal maturation, bright-field and vacuolar intrinsic fluorescence images of the same cells after fixation with glutaraldehyde were obtained by SCLIM. The bright-field image is shown on the left. cis-Golgi and TGN marker's image at one z section (at 43.64 s in Fig. 2 D, central left panel) and the vacuolar intrinsic fluorescence image (central right panel) are shown next. Scale bar, 1 µm. The TEM image of vacuolar membrane structure in thin section of plastic embedded cell is shown on the right. Scale bar, 1 µm. Fluorescence and TEM images show a well-overlapping vacuolar structure.

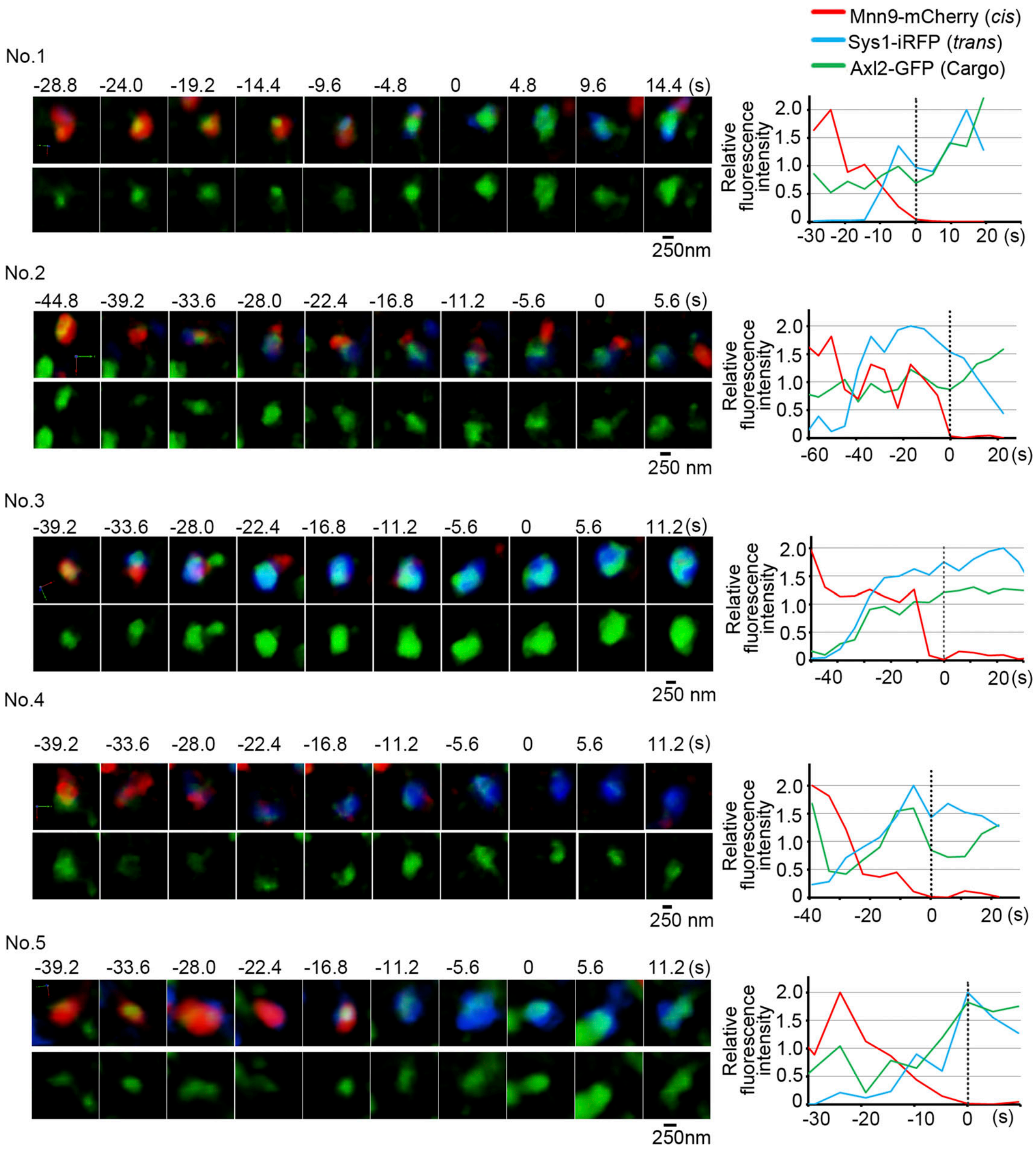

Figure S2. **Secretory cargo is transported from cis- to trans-Golgi while being maintained within a maturing cisterna.** *uso1-1* cells expressing heat-shock–inducible Axl2-GFP (cargo, green) and constitutive Mnn9-mCherry (cis-Golgi, red) and Sys1-iRFP (trans-Golgi, blue) were incubated at 37°C for 20 min and then shifted down to 24°C and observed by SCLIM. Five examples of 4D (3D time-lapse) observation of maturing cisternae are shown. 3D images were reconstructed from 16 optical slices 200 nm apart around the center of cell taken at 5.6-s intervals. The frame where Mnn9-mCherry signals disappeared was set as time 0. Merged (cargo, cis, and trans) and cargo images are shown on the left. Cargo was maintained within the Golgi cisterna, which matured from cis- to trans-Golgi. Relative fluorescence intensities of cargo, cis-Golgi, and trans-Golgi markers of the selected cisterna are shown on the right. Scale bar, 250 nm.

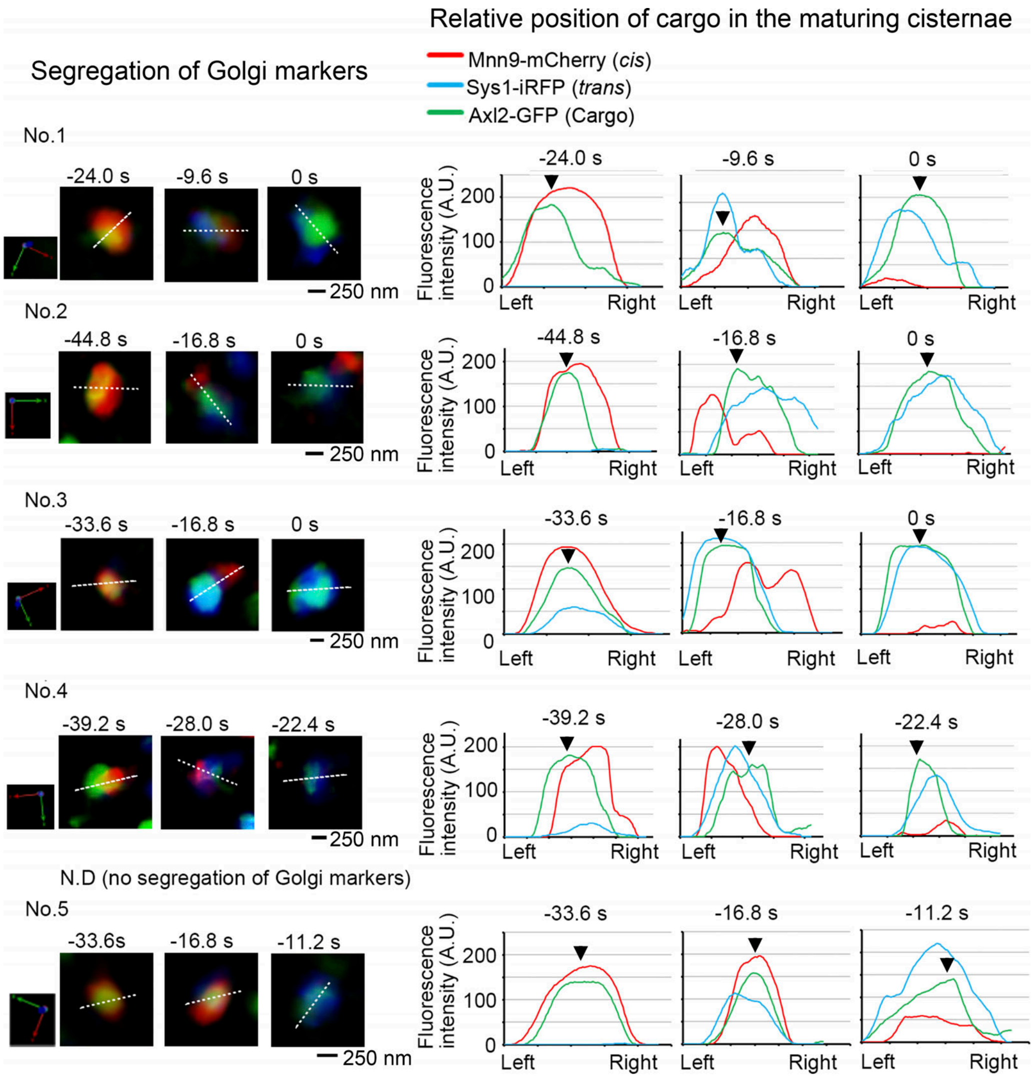

Figure S3. **Cargo moves from cis- to trans-Golgi zones within a cisterna during maturation.** Left panels show cargo localization within the maturing cisterna, which harbors distinct zones labeled with cis- and trans-Golgi markers, as shown in Fig. S3. Graphs on the right show relative fluorescence intensities of Axl2-GFP (cargo, green), Mnn9-mCherry (cis, red), and Sys1-iRFP (trans, blue) along the white lines in the maturing cisterna on the left at indicated times. No. 1 to No. 4 cells showed clear segregation of two Golgi markers during cisternal maturation, but No. 5 cell did not. Arrowheads indicate the peak positions of Axl2-GFP fluorescence intensities. Scale bar, 250 nm.

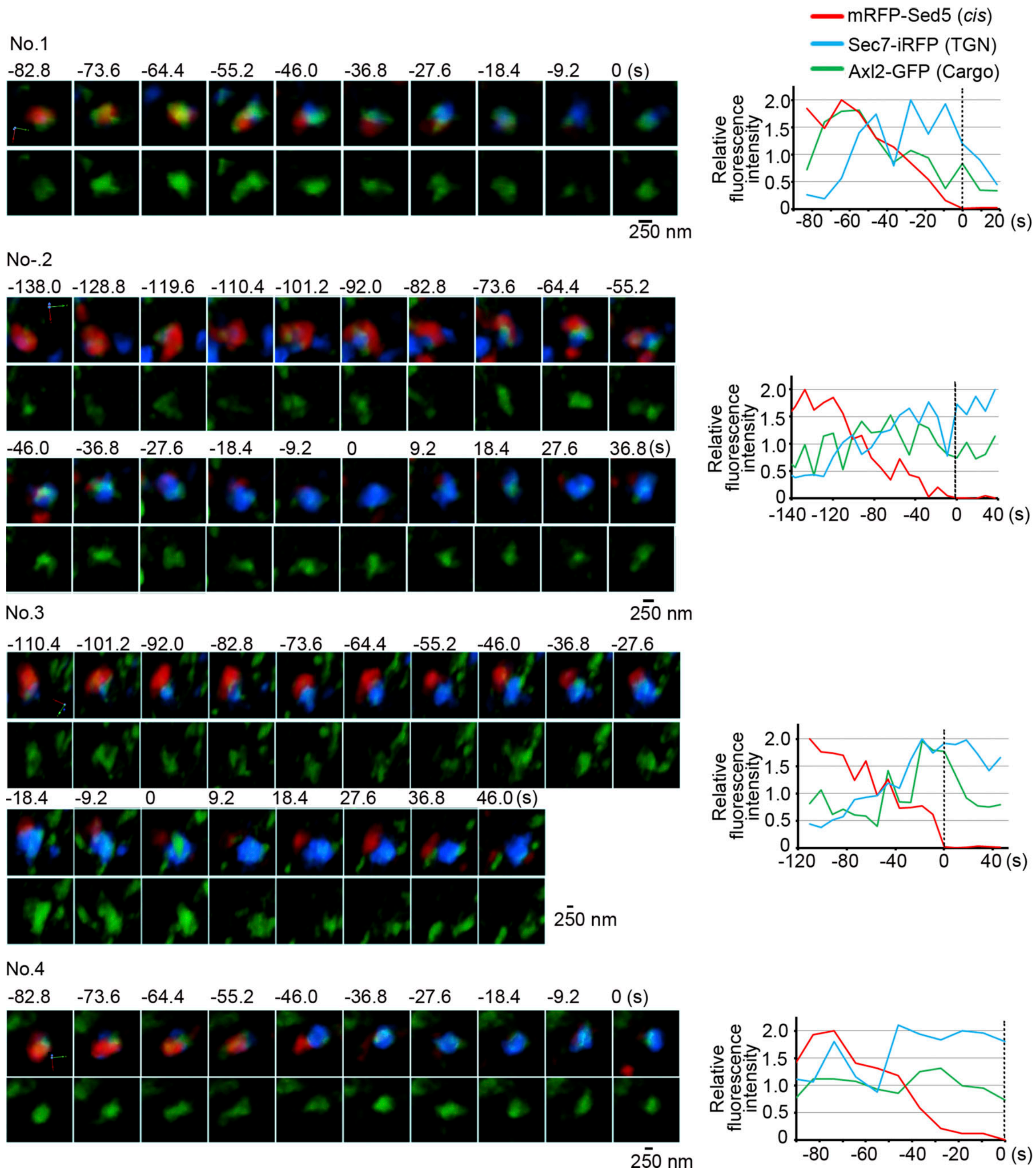

Figure S4. **Cargo is transported from cis-Golgi to the TGN while being maintained in a maturing cisterna.** *uso1-1* cells expressing heat-shock–inducible Axl2-GFP (cargo, green) and constitutive mRFP-Sed5 (cis-Golgi, red) and Sec7-iRFP (TGN, blue) were incubated at 37°C for 15 min and then shifted down to 24°C and observed by SCLIM. Four examples of 4D (3D time-lapse) observation of maturing cisternae are shown. 3D images were reconstructed from 26 optical slices 200 nm apart taken at 9.2-s intervals. Merged (cargo, cis, and TGN) and cargo images are shown. Relative fluorescence intensities of cargo, cis-Golgi, and TGN markers in the cisterna are shown on the right. Scale bar, 250 nm.

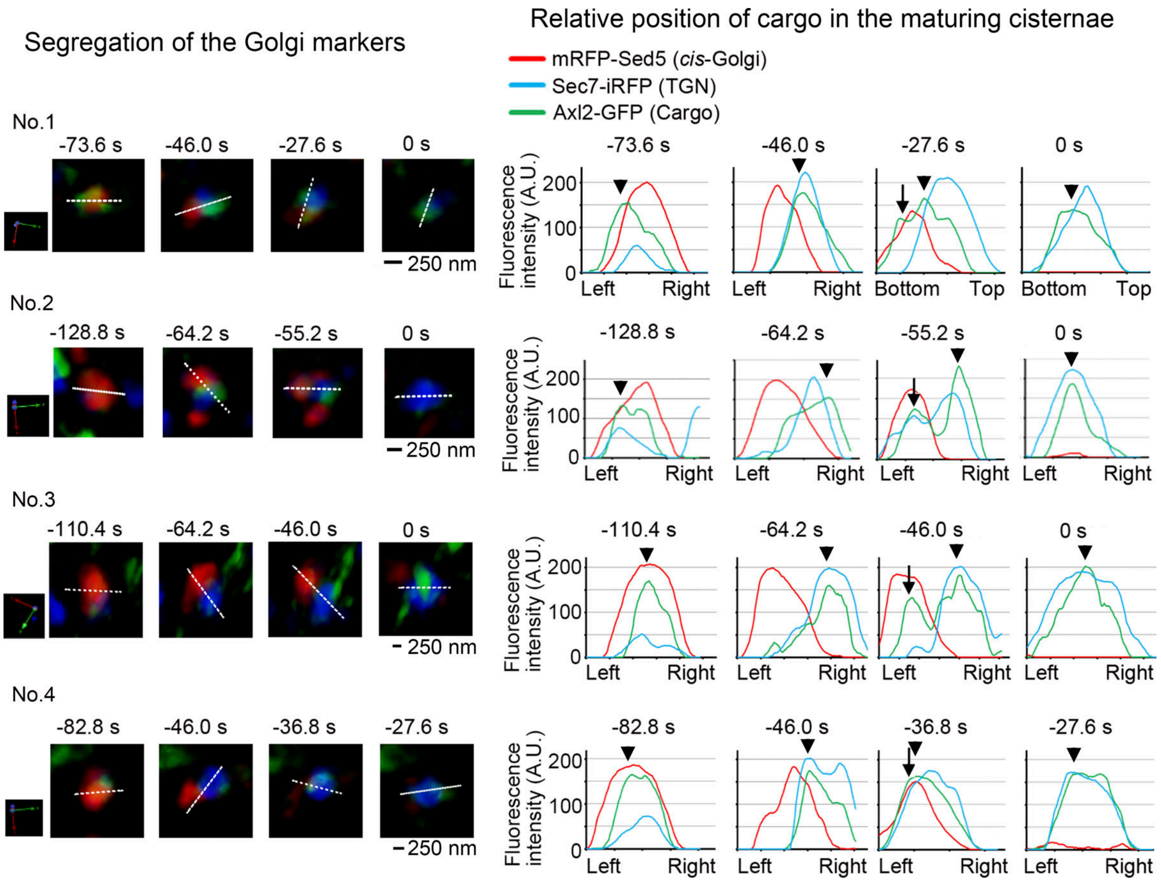

Figure S5. **Cargo moves from cis-Golgi to the TGN within a cisterna during maturation.** Left panels show cargo localization within the maturing cisterna, which harbors distinct zones labeled with cis-Golgi and TGN markers, as shown in Fig. S5. Graphs on the right show relative fluorescence intensities of cargo (green), cis-Golgi (red), and TGN (blue) markers along the white lines in the maturing cisterna on the left at indicated times. Note that cargo almost reached the TGN zone at the second time point (see arrowheads), but a small peak reappeared in the cis-Golgi zone at the third time point (see arrows). Cargo finally accumulated in the TGN zone (arrowheads at the fourth time point). Arrowheads indicate the peak positions of Axl2-GFP fluorescence intensities. Scale bar, 250 nm.

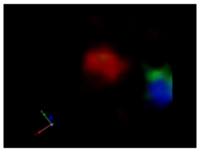

Video 1. **Three-color 4D observation of a WT cell expressing Mnn9-mCherry (cis-Golgi, red), Sys1-2xGFP (trans-Golgi, green), and Sec7-iRFP (TGN, blue).** A Golgi cisterna matures from cis- to trans-cisterna and then to the TGN.

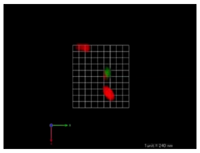

Video 2. **Multi-angle 3D reconstructed movie of a maturing cisterna (from cis to trans-Golgi) and a cis-Golgi cisterna by SCLIM and TEM.**

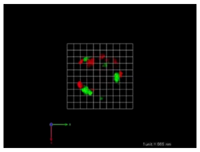

Video 3. **Multi-angle 3D reconstructed movie of a maturing cisterna (from cis-Golgi to the TGN) by SCLIM and TEM.** White arrowhead indicates a cis-Golgi cisterna labeled with mRFP-Sed5 (red) maturing to the TGN labeled with Sec7-GFP (green) in 3D reconstruction of SCLIM images.

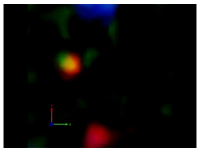

Video 4. **Three-color 4D movie of a Mnn9-mCherry positive cis-cisterna (red) with cargo Axl2-GFP (green) that matures into trans-Golgi cisterna labeled with Sys1-iRFP (blue).**

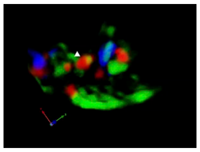

Video 5. **Another example of three-color 4D movie of a Mnn9-mCherry positive cis-cisterna (red) with cargo Axl2-GFP (green) that matures into trans-Golgi cisterna labeled with Sys1-iRFP (blue).** White arrowhead indicates a cis-Golgi cisterna (red) with cargo (green) maturing into trans-Golgi (blue). White arrow indicates another cis-Golgi cisterna (red) that approached and contacted with the ER, captured cargo, and then matured into trans-Golgi cisterna (blue).

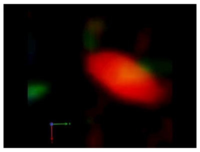

Video 6. **Three-color 4D movie of cis-Golgi cisterna labeled with Sed5-mRFP (red) with cargo Axl2-GFP (green) maturing to the TGN labeled with Sec7-iRFP (blue).**
